# Chemical Composition, Antioxidant, Anti-Diabetic, Anti-Acetylcholinesterase, Anti-Inflammatory, and Antimicrobial Properties of *Arbutus unedo* L. and *Laurus nobilis* L. Essential Oils

**DOI:** 10.3390/life12111876

**Published:** 2022-11-14

**Authors:** Samiah Hamad Al-Mijalli, Hanae Naceiri Mrabti, Hayat Ouassou, Rachid Flouchi, Emad M. Abdallah, Ryan A. Sheikh, Mohammed Merae Alshahrani, Ahmed Abdullah Al Awadh, Hicham Harhar, Nasreddine El Omari, Ahmed Qasem, Hamza Assaggaf, Naif Hesham Moursi, Abdelhakim Bouyahya, Monica Gallo, Moulay El Abbes Faouzi

**Affiliations:** 1 Department of Biology, College of Sciences, Princess Nourah bint Abdulrahman University, P.O. Box 84428, Riyadh 11671, Saudi Arabia; 2 Laboratory of Pharmacology and Toxicology, Bio Pharmaceutical and Toxicological Analysis Research Team, Faculty of Medicine and Pharmacy, University Mohammed V in Rabat, Rabat BP 6203, Morocco; 3 Faculty of Sciences, University Mohammed First, Boulevard Mohamed VI BP 717, Oujda 60000, Morocco; 4 Laboratory of Microbial Biotechnology and Bioactive Molecules, Science and Technologies Faculty, Sidi Mohamed Ben Abdellah University, Fez BP 2202, Morocco; 5 Department of Science Laboratories, College of Science and Arts, Qassim University, Ar Rass 51921, Saudi Arabia; 6 Biochemistry Department, Faculty of Science, King Abdulaziz University, Jeddah 21589, Saudi Arabia; 7 Department of Clinical Laboratory Sciences, Faculty of Applied Medical Sciences, Najran University, Najran 61441, Saudi Arabia; 8 Laboratory of Materials, Nanotechnology and Environment LMNE, Faculty of Sciences, Mohammed V University in Rabat, Rabat BP 1014, Morocco; 9 Laboratory of Histology, Embryology, and Cytogenetic, Faculty of Medicine and Pharmacy, Mohammed V University in Rabat, Rabat 10000, Morocco; 10 Department of Laboratory Medicine, Faculty of Applied Medical Sciences, Umm Al-Qura University, Makkah 21955, Saudi Arabia; 11 King Fahad Armed Forced Hospital, Jeddah 23311, Saudi Arabia; 12 Laboratory of Human Pathologies Biology, Department of Biology, Faculty of Sciences, Mohammed V University in Rabat, Rabat BP 6203, Morocco; 13 Department of Molecular Medicine and Medical Biotechnology, University of Naples Federico II, Via Pansini 5, 80131 Naples, Italy

**Keywords:** *Laurus nobilis*, *Arbutus undeo*, volatile compounds, anti-diabetic, anti-inflammatory, antimicrobial

## Abstract

The objectives of this work were to determine the phytochemical composition and antioxidant, anti-diabetic, antibacterial, anti-inflammatory, and anti-acetylcholinesterase properties of *Arbutus unedo* L. and *Laurus nobilis* L. EOs. The antioxidant effects were estimated using four complementary methods. In addition, the anti-diabetic activity was assessed by targeting three carbohydrate-hydrolyzing enzymes, namely α-amylase, α-glucosidase, and lipase. The anti-inflammatory and anti-acetylcholinesterase effects were evaluated by testing the inhibitory potential of both plants on lipo-oxygenase and acetylcholinesterase (AChE), respectively. The antimicrobial activity of these oils was evaluated using disc-diffusion, minimum inhibitory concentration (MIC), and minimum lethal concentration (MLC) tests. The chemical composition of *L. nobilis* essential oil (EO) was dominated by eucalyptol (36.40%), followed by α-terpineole (13.05%), α-terpinyl acetate (10.61%), linalool (10.34%), and northujane (5.74%). The main volatile compounds of *A. unedo* EOs were decenal (13.47%), α-terpineol (7.8%), and palmitic acid (6.00%). *L. nobilis* and *A. unedo* EOs inhibited α-amylase with IC_50_ values of 42.51 ± 0.012 and 102 ± 0.06 µg/mL, respectively. Moreover, both oils inhibited the activity of α-glucosidase (IC_50_ = 1.347 ± 0.021 µg/mL and IC_50_ = 76 ± 0.021 µg/mL) and lipase (IC_50_ = 21.23 ± 0.021 µg/mL and IC_50_ = 97.018 ± 0.012 µg/mL, respectively). In addition, *L. nobilis* EO showed an anti-AChE activity (IC_50_ = 89.44 ± 0.07 µg/mL) higher than that of *A. unedo* EO (IC_50_ = 378.57 ± 0.05 µg/mL). Regarding anti-inflammatory activity, in vitro assays showed that *L. nobilis* significantly inhibits (IC_50_ = 48.31 ± 0.07 μg/mL) 5-lipoxygenase compared to *A. unedo* (IC_50_ = 86.14 ± 0.05 μg/mL). This was confirmed in vivo via a notable inhibition of inflammation recorded after 6 h of treatment in both plants at a dose of 50 mg/kg. The microbiological results revealed that EOs from both plants inhibited the growth of all tested organisms except *P. aeruginosa*, with the highest antimicrobial effect for *L. nobilis*. The results of these tests showed that these two plants possess remarkable biological and pharmacological properties, explaining their medicinal effects and suggesting them as promising sources of natural drugs.

## 1. Introduction

Despite the current emphasis on synthetic pharmaceuticals, medicinal plants have always been and will continue to be the primary source of drugs [1]. Even today, medicinal plants are believed to be the main source of health care for up to 80% of the world’s population, most of whom live in developing countries [2]. Plant extracts, especially essential oils (EOs), include several phytochemicals with diverse physiological effects on the body [3,4,5,6,7,8,9,10,11,12,13]. EOs are mixtures of molecules extracted from plants primarily through steam distillation, which collects the major aromatic compounds such as terpenoids and phenolic compounds [13,14].

Indeed, different methods of extraction have been developed recently for the isolation of secondary metabolites [15,16,17,18]. The secondary metabolites have many medical uses, including antioxidants, antibacterial, antifungal, antiviral, anticancer, anti-inflammatory, and antiprotozoal [1,19,20,21,22].

Antioxidants have recently gained scientific attention following recent studies demonstrating various health benefits, including anti-inflammatory and anti-aging properties [23]. Moreover, free radicals are considered pathophysiological agents. Hence, antioxidant intake protects against oxidative stress by preventing the formation of reactive species [24]. Experimental evidence shows that high levels of antioxidants may be beneficial in inhibiting several types of free radical damage related to the development of diabetes mellitus (DM) [25,26,27]. Medicinal plants are widely popular in many countries and are used in alternative medicine and as supplementary foods [28,29].

With the recent spread of microbial infections and the growing concern that antibiotics may not be able to inhibit the growth of antibiotic resistant pathogens in the future, there is an urgent need for new sources of drugs such as natural products rich in antimicrobial compounds [30,31]. Many studies have been conducted on the antimicrobial properties of extracts and EOs from medicinal plants or their isolated compounds such as phenolics, flavonoids, lactones, terpenes, naphtoquinones, or alkaloids [32,33,34,35,36]. Some of these phytochemicals were identified after discovering antibacterial activity in the plant, and this process is called bioguided isolation and determination of phytochemicals [13]. In addition, recent studies have been able to decipher the molecular mechanisms through which plants or their active ingredients act. Indeed, natural antimicrobial molecules can act at several levels; sub-cellular (cell wall and membranes), cellular (intracellular signaling of microbes), and molecular (DNA replication and transcription, as well as protein synthesis) [37].

On the other hand, scientific research on the management of DM has attracted worldwide attention because it is a growing global health problem and often significantly increases the risk of many cardiovascular issues, such as coronary artery disease with chest discomfort, heart attacks, strokes, arterial constriction, and nerve damage [38]. Sulfonylureas, α-glucosidase inhibitors, biguanides, and thiazolidinediones are some of the currently available anti-diabetic drugs that are often used to manage hyperglycemia. However, the progression of diabetes complications is not dramatically changed by these medications. Due to unfavorable clinical circumstances and high risks of subsequent failure, they are only sometimes used. It is, therefore, crucial to seek more effective antidiabetic treatments with fewer adverse effects [39]. Today, there are more than 410 medicinal plants with antidiabetic activities whose effectiveness in the treatment of hyperglycemia, a metabolic disorder, has been scientifically proven in various ways (in vitro, in vivo, and in clinical studies) [40].

*Arbutus unedo* L. (Ericaceae), is widely distributed in Mediterranean countries, such as Morocco, Algeria, Tunisia, Spain, Portugal, France, Syria, Turkey, Greece, and Croatia [41]. In Morocco, it is commonly called “sasnou” or “Bakhano”. It has been used in folk medicine as an antiseptic, anti-diabetic, anti-hypertensive, diuretic, and laxative [42,43,44,45]. *A. unedo* is already known as a good source of organic acids and antioxidants, including phenolic compounds, vitamins (C and E), and carotenoids [44,46]. *Laurus nobilis* L. (Lauraceae), is an evergreen tree cultivated in many warm regions of the world, especially in Mediterranean countries such as Morocco, Algeria, Spain, Portugal, Turkey, and Greece [47]. It has long been used to treat diabetes, rheumatism, dermatitis, stomach problems, snakebites, and migraines [48].

Therefore, the aims of this study were to determine the chemical profile and investigate the antioxidant, anti-diabetic, anti-inflammatory, anti-acetylcholinesterase, and antimicrobial properties of *Arbutus unedo* EO (AUEO) leaves and *Laurus nobilis* EO (LNEO) leaves. Although the biological activities of these two plants have been previously studied, certain biological tests such as the anti-inflammatory effects remain to be developed. Moreover, the two studied plants were collected from a region where they have yet been studied.

## 2. Material and Methods

### 2.1. Chemicals and Reagents

2,2-diphenyl-1-picrylhydrazyl (DPPH), 2,2-azino-bis-3-ethylbenzothiazoline-6-sulfonic acid (ABTS), 6-hydroxy-2,5,7,8-tetramethylchroman-2-carboxylic acid (Trolox), and ascorbic acid were purchased from Sigma-Aldrich (Saint-Quentin-Fallavier, France). Lipoxygenase (5-LOX) and linolenic acid were purchased from Sigma-Aldrich (St. Louis, MO, USA). Mueller–Hinton Agar (MHA), Sabouraud dextrose agar (SA), Tryptone Soy agar, dimethyl sulfoxide (DMSO), and chloramphenicol were purchased from Biokar (Beauvais, France). All the other reagents were of analytical grade.

### 2.2. Collection of Medicinal Plants

Leaves of both (2 kg) used medicinal plants *(A*. *unedo* and *L. nobilis)* were collected from the region of Taza, Morocco. The botanical identification of both plants was carried out at the botany department of the Scientific Institute (Mohammed V University in Rabat). RAB10549 and RAB10143 are the attributed voucher specimens for *A. unedo* and *L. nobilis*, respectively.

### 2.3. Isolation of Essential Oils

Essential oils of *A. unedo* and *L. nobilis* leaves were isolated by a hydrodistillation procedure using Clevenger apparatus. The dry leaves are deposited in the Clevenger apparatus with water for 3 h. The volatile compounds (essential oils) are vaporized with the water and subsequently separated after cooling by density difference.

### 2.4. Identification of Chemical Compounds

The analytical technique of gas chromatography-mass spectrometry (GC-MS) was used to characterize and identify the chemical compounds of AUEO and LNEO as published by Al-Mijalli et al. [49].

A 5% phenylmethyl silicone HP-5MS capillary column (30 m × 0.25 mm × film thickness of 0.25 μm) was used in GC. The temperature of the column was increased from 50 °C for 5 min to 200 °C with a 4 °C/min rate. The used carrier gas was helium with a 1.5 mL/min flow rate and a split mode (flow: 112 mL/min, ratio: 1/74.7). The hold time was 48 min, and the injector and detector were both at 250 °C. MS operating conditions functionned at 70 eV ionization voltage, 230 °C ion source temperature, and a 35–450 (*m*/*z*) scanning range. The identification of chemical composition was done by comparing the MS spectra with the library and matching the Kovats index (Library of NIST/EPA/NIH MASS SPECTRAL LIBRARY Version 2.0, 1 July 2002). Moreover, an internal normalization of the total area of peaks detected in each chromatogram was done for the quantification of each compound.

### 2.5. Determination of Antioxidant Activity

#### 2.5.1. Hydroxyl Radical Scavenging Assay

Hydroxyl radical scavenging activity was carried out according to a slightly modified method by Basak et al. [48]. The hydroxyl radicals produced by the Fe^3+^/ascorbate/EDTA/H_2_O_2_ system were measured to assess the hydroxyl radical scavenging activity. Deoxyribose is attacked by the hydroxyl radical, which causes the production of compounds that react with thiobarbituric acid (TBARS). A volume of 100 µL of each sample (1000 µg/mL) was added to a reaction mixture containing 100 µL of 3.0 mM deoxyribose, 100 µL of 0.1 mM FeCl_3_, 100 µL of 0.1 mM EDTA, 100 µL of 0.1 mM ascorbic acid, 100 µL of 1 mM H_2_O_2_, and 20 mM phosphate buffer (pH = 7.4) in a final volume of 1.0 mL. Then, the absorbance was measured at 532 nm against a blank containing deoxyribose and buffer. The percentage inhibition (I) of deoxyribose degradation was calculated as follows:

% I = (A_0_ − A_1_/A_0_) × 100

where A_0_ is the absorbance of the control reaction and A_1_ is the absorbance of the test compound.

#### 2.5.2. Inhibition of Superoxide Radical Assay

Superoxide radical generation by the xanthine/xanthine oxidase system was determined according to Basak et al. [48]. Briefly, a 100 µL of each sample (1000 µg/mL) was added to a reaction mixture containing 100 μL of 2 nM xanthine, 100 μL of 12 nM NBT, 100 μL of 1.0 U/mL xanthine oxidase, and 0.1 M phosphate buffer (pH = 7.4), making a final volume of 2.0 mL. After incubating the mixture at 25 °C for 10 min, the percent inhibition of superoxide anion was calculated using the following equation:

% Inhibition = (A_0_ − A_1_/A_0_) × 100

where A_0_: the absorbance of the control and A_1_: the absorbance of the samples.

#### 2.5.3. DPPH Assay

The method (DPPH), described by Basak et al. [48], follows the bleaching of a purple methanol solution of DPPH. Briefly, 50 µL of the essential oil (1000 µg /mL) was added to 5 mL of a 0.004% solution of DPPH in methanol. The absorbance was measured at 517 nm after 30 min of incubation at dark conditions. The DPPH radical scavenging activity was calculated using the following formula:

DPPH inhibition percentage = (A_0_ − A_1_/A_0_) × 100

where A_0_ is the absorbance of the control and A_1_ is the absorbance of the presence of samples.

#### 2.5.4. Lipid Peroxidation Inhibition Assay

Non-enzymatic lipid peroxidation assay was realized according to the procedure described by Basak et al. [48], with minor changes. The reaction mixture contained 100 μL of each sample (1000 µg/mL), 100 μL of supernatant, 20 μL of 1 mM FeCl_3_, and 20 μL of 1 mM ascorbic acid to induce hydroxyl radical generation. After a 1 h incubation period at 37 °C, the extent of lipid peroxidation was measured by the TBA reaction. After cooling, 2.5 mL of *n*-butanol was added and the samples were centrifuged at 3500 rpm for 5 min. The absorbance was read at 532 nm. The percentage inhibition of activity was calculated using the formula:

% inhibition = (A_0_ − A_1_/A_0_) × 100

In this equation, A_0_ is the absorbance of the control (without sample) and A_1_ is the absorbance of the samples.

### 2.6. In Vitro Anti-Diabetic Assay

The potential of oils of *A. unedo* and *L. nobilis* to inhibit the enzymatic activity of α-amylase and α-glucosidase was assessed according to previously published research [50,51], and the assay of the lipase inhibitory activity was conducted according to the method described by Hu et al. [52].

### 2.7. Anti-Acetylcholinesterase Activity

Inhibition of acetylcholinesterase (AChE) activity was measured using an adaptation of the method described by Ingkaninan et al. [53], with some modifications. Briefly, 10 µL solution of EOs in Tween 80 (0.5% *v*/*v*) at different concentrations and 25 µL of AChE enzyme were mixed with 0.1 M of TrisHCl buffer at 0.1 M concentration and pH 8. The solutions were incubated for 15 min at room temperature. After incubation, 10 µL of a solution of acetylthiocholine iodide (ASCh) of 0.5 mM concentration and 10 µL of 5,5′-dithio-bis-2-nitrobenzoic acid (DTNB) of 3 mM concentration were added. The absorbance of the mixture was measured at 412 nm in a UV-visible spectrophotometer.

### 2.8. In Vitro Anti-Inflammatory Assays

The in vitro anti-inflammatory effect of AUEO and LNEO were assessed by the 5-Lipoxygenase (5-LOX) inhibitory activity, according to the previously published method [48]. Briefly, 20 µL of EOs and 20 µL of 5-LOX from Glycine max (100 U/mL) were pre-incubated with 200 µL of phosphate buffer (0.1 M, pH 9) at room temperature for 5 min. Then, 20 µL of linolenic acid (4.18 mM in ethanol) was added in order to start the reaction, which was followed for 3 min at 234 nm.

### 2.9. In Vivo Anti-Inflammatory Assay

The anti-inflammatory effects were investigated using a rat model of carrageenan-induced paw edema (Rege et al. [54]). Briefly, Wistar rats (150 to 180 g) were fasted for 18 h and then randomly divided into eight groups (n = 6 per group). The groups of rats received different concentrations of the studied drugs (AUEO and LNEO, (1:1)) (50 and 100 mg/kg). The control group received distilled water while the other groups received indomethacin (10 mg/kg) as the reference drug. After 60 min, all rats were injected subcutaneously with carrageenan solution (0.05 mL of 1% carrageenan suspended in 0.9% NaCl) into the subplantar region of the left hind paw. The paw volumes of the tested rats were recorded using a LE 7500 digital plethysmometer controlled by SeDaCOM software before the injection of carrageenan (*V*_0_), and after the carrageenan injection at three different times 1 h, 3 h, and 6 h (*V_t_*). The anti-inflammatory effect is calculated using the following equation:

$$\%\;inhibition=\frac{(V_{t}-V_{0})\;control-(V_{t}-V_{0})\;treated\;group}{(V_{t}-V_{0})\;\;control}\times 100$$

### 2.10. Antimicrobial Activity

#### 2.10.1. Tested Microorganisms

Microorganisms were used to test for antimicrobial activity, including four Gram-negative bacterial strains: *Escherichia coli* (ATCC 25934), *Proteus mirabilis* (ATCC 25933), *Salmonella typhimurium* (ATCC 700408), and *Pseudomonas aeruginosa* (ATCC 27853); three Gram-positive bacterial strains: *Bacillus subtilis* (ATCC 6633), *Listeria monocytogenes* (ATCC 13932); one yeast strain: *Candida albicans* (clinical isolate); and two fungal strains: *Trichophyton rubrum* (clinical isolate) and *Aspergillus niger* (food-spoilage isolate). Microorganisms were generously provided by the Laboratory of Microbial Biotechnology and Bioactive Molecules, Science and Technologies Faculty, Sidi Mohamed Ben Abdellah University, Morocco. All isolates were preserved at −20 °C until used. The bacterial strains were sub-cultured on MHA medium (brought from the Laboratory of Microbial Biotechnology and Bioactive Molecules) at 37 °C for 18 h. Yeast and fungal isolates were sub-cultured on SA medium (brought from the Laboratory of Microbial Biotechnology and Bioactive Molecules) at 25 °C for 48 h and five days, respectively.

#### 2.10.2. Inoculum Preparation

Fresh microbial cultures were adjusted to 0.5 McFarland Standard’s turbidity. To make the McFarland standard, 99.5 mL of 1% (vol/vol) sulfuric acid was combined with 0.5 mL of a solution of barium chloride dihydrate (BaCl_2_•2H_2_O), which has a weight-to-volume ratio of 1.175%. The adjusted sample of the suspension contains about 10^8^ colony forming units (CFU/mL) for bacteria, approximately 10^6^ CFU/mL for yeast, and around 10^4^ spore forming/mL for fungi [13,55].

The adjustment of the microbial suspensions for reactivation was carried out by inoculating MHA medium with a loopful of the frozen (−20 °C) stock and incubating it at 37 °C for 24 h for bacteria and for 48 to five days for yeast and fungi. The antimicrobial testing was conducted using modified microbial innocula.

#### 2.10.3. Disc-Diffusion Assay

The antimicrobial screening of AUEO and LNEO was performed using the disc diffusion technique according to previously reported methods [56,57]. The culture suspension was inoculated on MHA medium for bacteria and SA medium for yeast and fungal strains. Sterile paper discs, 6 mm in diameter sterile paper discs soaked in 10 µL of each EO (combined with 5% DMSO) were then placed on each plate. The positive control for bacteria was chloramphenicol (30 µg), the positive control for yeasts and fungi was nystatin (100 I.U.), and the negative control was DMSO (10 µL; 5%). The bacterial plates were incubated at 37 °C for 24 h, while the fungal and yeast plates were incubated at 25 °C for 48 and 72 h, respectively. The widths of the inhibitory zones were measured in millimeters after incubation, and the findings were expressed as the mean and standard deviation of three replicates.

#### 2.10.4. Determination of Minimum Inhibitory Concentration

The minimum inhibitory concentration (MIC) was determined using the microtube dilution method as described in [58]. Briefly: in sterile microtubes consisting of 100 μL of MHA broth (for bacteria) or SA broth (for yeast and fungi) and containing 5% Tween 80 (emulsifier), a decreasing concentration of EOs from 4% to 0.0625% (*v*/*v*) were prepared. Then, 5 µL of the adjusted microbial suspension (adjusted to 0.5 McFarland as previously described) were added to each tube. Another set of tubes containing liquid media without EOs and inoculated with the adjusted microbial suspensions, and un-inoculated tubes containing liquid media plus EOs served as negative controls. A microtube set containing serial dilutions of chloramphenicol and nystatin instead of EOs served as positive controls. After incubation, microtubes with low concentrations and without visible microbial growth were considered the MIC [57].

#### 2.10.5. Determination of Minimum Lethal Concentration

The minimum lethal concentration (MLC) was carried out following the MIC test. In aseptic conditions, 5 µL of each tube that had no apparent growth on the MIC test was poured onto plates containing MHA (for bacteria) or SA agar (for yeast and fungi) and incubated at 35–37 °C for 24 h for bacteria, or at 25 °C for 48 and 72 h for yeast and fungi, respectively. Then, incubated plates were inspected. The lowest concentration of EO at which a microorganism can be killed (no visible growth) was deemed the MLC [59,60].

### 2.11. Statistical Analysis

The findings of each experiment were run in triplicate, and they are presented as mean ± standard deviations (SD). The data were analyzed using IBM SPSS Statistics for Windows, Version 21.0 Armonk, NY, USA, and one-way ANOVA in addition to the Tukey test were used to compare means. When *p* < 0.05, the differences between the means were deemed significant.

## 3. Results

### 3.1. Chemical Composition

Chemical analyses of AUEO and LNEO constituents, the percentage content of each compound, elution order, structural subclass, and retention index are presented in Table 1. Chemical analysis of EOs from *A. undeo* and *L. nobilis* found 15 and 18 chemical compounds, which make up 53.33% and 89% of the total composition of these oils, respectively.

The AUEO was characterized by a high level of decenal (13.47%) accompanied by other constituents with variable content, such as α-terpineol (7.8%) and palmitic acid (6.00%). For LNEO, monoterpenes constituted the largest class of terpenes (72.18% oxygenated monoterpenes and 4.88% monoterpene hydrocarbons). Moreover, the EO of this plant was dominated by eucalyptol (36.40%), followed by α-terpineole (13.05%), α-terpinyl acetate (10.61%), linalool (10.34%), and *β*-thujene (5.74%).

### 3.2. Antioxidant Activity

In this study, four different in vitro assays, namely hydroxyl radical, superoxide radical, lipid peroxidation, and DPPH radical scavenging activity, were adopted to determine the antioxidant properties of AUEO and LNEO. The IC_50_ values in Table 2 showed significant differences (*p <* 0.05) between the antioxidant activities of AUEO, LNEO, and butylated hydroxytoluene (BHT), used as a positive control. Based on the antioxidant potency achieved using the hydroxyl radical (OH) test, it was found that the IC_50_ value of AUEO (0.527 ± 0.01 μL/mL) and LNEO (0.354 ± 0.02 μL/mL) exhibited the highest inhibitory activity against hydroxyl radicals compared to the antioxidant BHT (12.027 ± 0.01 μL/mL).

In this sense, LNEO has been reported to be able to scavenge hydroxyl radicals generated by an in vitro Fe^3+/^ascorbate/EDTA/H_2_O_2_ system [48]. Moreover, AUEO and LNEO were found to scavenge superoxide with an IC_50_ of 0.275 ± 0.07 μL/mL and 0.133 ± 0.01 μL/mL, respectively. In comparison, the standard BHT had a superoxide radical scavenging activity of 43.307 ± 0.001 μL/mL. For the lipid peroxidation method, the IC_50_ values were found to compare favorably (0.207 ± 0.03 μL/mL and 0.101 ± 0.05 μL/mL for AUEO and LNEO, respectively) with that obtained by BHT (IC_50_ = 2.022 ± 0.031 μL/mL). In addition, strong DPPH free radical scavenging activity was recorded for AUEO and LNEO with IC_50_ of 0.711 ± 0.04 μL/mL and 0.489 ± 0.07 μL/mL, respectively. These results may be compared to BHT showing an IC_50_ equal to 21.057 ± 0.051 μL/mL.

### 3.3. Anti-Diabetic Activity

One of the most widely used approaches to reducing postprandial hyperglycemia and managing diabetes is to inhibit digestive enzymes such as α-amylase and α-glucosidase [61]. Table 3 shows the results of the α-amylase, α-glucosidase, and pancreatic lipase inhibition activities of AUEO and LNEO. Indeed, AUEO and LNEO were discovered to have a significant inhibitory effect on the α-amylase enzyme, with IC_50_ values of 102 ± 0.06 µg/mL and 42.51 ± 0.012, respectively. The results were compared to those of acarbose, a reference antidiabetic, which showed an IC_50_ of 32.14 ± 0.016 µg/mL. Interestingly, LNEO showed a much higher α-glucosidase inhibitory activity (IC_50_ = 1.347 ± 0.021 µg/mL) than that of the control (IC_50_ = 22.0 ± 0.005 µg/mL), while AUEO showed an activity of 76 ± 0.021 µg/mL against the same enzyme. With respect to lipase inhibition, AUEO and LNEO inhibited pancreatic lipase activity with IC_50_ values of 97.018 ± 0.012 µg/mL and 21.23 ± 0.021 µg/mL, respectively, when compared to the reference compound, orlistat, which had lipase inhibitory activity with an IC_50_ value of 14.12 ± 0.023 μg/mL (Table 3).

### 3.4. Anti-Acetylcholinesterase Activity

Acetylcholinesterase (AChE) is an enzyme implicated significantly in neurodegenerative diseases, particularly Alzheimer’s disease (AD). Moreover, its inhibition constitutes a major therapeutic and preventive pathway. In this work, the inhibitory effects of the two EOs and the control (rivastigmine) were tested against AChE. The results are expressed in IC_50_ and presented in Table 4. As indicated in the table, LNEO showed a greater inhibitory effect (IC_50_ = 89.44 ± 0.07 µg/mL) than AUEO (IC_50_ = 378.57 ± 0.05 µg/mL); rivastigmine showed high activity (IC_50_ = 2.24 ± 0.03 µg/mL).

### 3.5. Anti-Inflammatory Activity

A large number of aromatic medicinal plant species contain various bioactive compounds with beneficial health properties, especially anti-inflammatory effects. Therefore, using EOs as natural additives is a good approach to prevent abnormal inflammation [62,63].

In this context, the determination of anti-inflammatory effect of the AUEO and LNEO in our work was investigated both in vitro, using 5-LOX enzyme, and in vivo using carrageenan-induced mouse paw edema.

Both EOs tested showed more or less promising inhibitory activity of 5-LOX with IC_50_ values of 86.14 ± 0.05 μg/mL for AUEO and 48.31 ± 0.07 μg/mL for LNEO in comparison with the control (quercetin), which showed an IC_50_ value of 17.59 ± 0.01 μg/mL (Table 5). Moreover, the results of an in vivo experiment showed a significant inhibition of carrageenan-induced hind paw edema volume after 6 h of treatment with these oils at a dose of 50 mg/kg, with a percentage inhibition of 58.82% for AUEO and 70.59% for LNEO compared to the anti-inflammatory drug, indomethacin (72.55%), used as a positive control (Table 6).

### 3.6. Antimicrobial Activity

The disc diffusion method was carried out to evaluate the antimicrobial activity of EOs from AUEO and LNEO. The results are shown in Table 7. The findings revealed that EOs from both plants had significant antimicrobial activity against all tested microorganisms compared to the conventional antibiotics; chloramphenicol for bacteria and nystatin for yeasts and fungi (ANOVA, *p <* 0.05). It should be noted that, although superior to chloramphenicol, *P. aeruginosa* showed the weakest activity (in vitro). In general, the Gram-positive bacteria recorded the highest mean zone of inhibition (varying between 15.4 ± 0.2 and 19.3 ± 0.2 mm) compared to Gram-negatives (varying between 8.0 ± 0.0 and 16.2 ± 0.1 mm), and the mean inhibition zones of yeasts (ranging from 16.2 ± 0.2 to 19.8 ± 0.3 mm) were higher than the fungal strains (ranging from 13.0 ± 0.3 to 16.2 ± 0.2 mm), although antibiotics remained the most effective (Table 7).

The results of the MIC and MLC tests are shown in Table 8. According to the MIC values, the lowest concentration of AUEO and LNEO inhibiting a microbe’s visible growth ranged between 2 and 1% for the tested Gram-negative bacteria (except for *P. aeruginosa* which was >4%), 1 and 0.5% for all Gram-positive bacteria tested, 0.5 and 0.25% for yeasts (*Candida albicans*), and 2 and 1% for the tested fungal strains, respectively. On the other hand, the MLC values are represented, in this study, by the lowest concentration of the EOs killing the test organism under in vitro conditions.

In the current study, the MLC values of AUEO and LNEO ranged between 2 and 1% (except with *P. aeruginosa* which was >4%); the Gram-positive bacteria ranged between 2–1 and 1%, respectively. Interestingly, *P. aeruginosa* was not susceptible to AUEO and LNEO.

## 4. Discussion

### 4.1. Chemical Composition

The analysis of phytochemical composition is an indispensable tool in the development of active ingredients derived from plant tissues. In our study, several natural substances have been identified from the studied plants that could be responsible for their various biological activities.

Indeed, the AUEO was characterized by a high rate of decenal (13.47%), accompanied by other constituents with variable content, such as α-terpineol (7.8%) and palmitic acid (6.00%). It should also be noted that the EO of the same species in Algeria showed a significant amount of some major chemical constituents such as palmitic acid (35.2%), linoleic acid (18.8%), and 2,6-di-tert-butyl-p-cresol (6.2%) [64]. Likewise, AUEO from Turkey showed a significant amount of various chemical constituents. Indeed, thirty-seven constituents were characterized as present, including (E)-2-decenal (12.0%), α-terpineol (8.8%), hexadecanoic acid (5.1%), and (E)-2-undecenal (4.8%) as the major constituents [65]. This variation in chemical profiles of essential oils could be attributed to several factors, such as harvest season, plant age, soil composition, and geographical variation [50,51,66,67].

In our work, from a practical point of view, our results are comparable to other studies on EOs extracted from Moroccan *L. nobilis* (northern Morocco) [47], identifying about 26 compounds, with the predominance of eucalyptol (52.43%), followed by α-terpinyl acetate (8.96%), sabinene (6.13%), limonene (5.25%), β-pinene (3.72%), linalool (3.14%), terpinene-4-ol (2.56%), α-terpinene (2.12%), β-terpineol (1.56%), bornyl acetate (1.89%), α-phellandrene (1.28%), myrcene (1.13%), camphene (1.05%), p-cymene (0.94%), α-terpinene (0.98%), and eugenol (0.56%).

Numerous studies have evaluated the chemical composition of LNEO in different countries. In Argentina, Lira et al. [68] showed a predominance of eucalyptol (45.1%), then linalool (11.9%), sabinene (9.3%), α-terpinyl acetate (8.0%), and methyl eugenol (2.8%). In Bulgaria, Fidan et al. [69] revealed the presence of eucalyptol (41.0%), followed by α-terpinyl acetate (14.4%), sabinene (8.8%), methyl eugenole (6.0%), β-linalool (4.9%), and α-terpineol (3.1%), and in Iran, Mohammadreza [70] identified a preponderance of eucalyptol (55.80%), α-terpinyl acetate (15.14%), terpinene-4-ol (5.27%), α–pinene (5.26%), p-cymene (2.70%), linalool (1.40%), and terpinene-4-yla cetate (1.13%). In Turkey, Dadalioǧlu and Evrendilek [71] found eucalyptol (60.72%), α-terpinene (12.53%), sabinene (12.12%), and α-pinene (6.11%) as major constituents.

In Algeria, Nabila et al. [72] revealed chemical variations of LNEO with a predominance of oxygenated monoterpenes (59%), eucalyptol (30.1%), and α-terpynil acetate (21.6%) as major compounds. The second-most-abundant class was phenylpropanoids (18.7%), which were made up of methyl eugenol (16.9%), elemicin (0.9%), and apiole (0.9%).

### 4.2. Antioxidant Activity

The evaluation of the antioxidant activity was carried out using several in vitro tests. This allows one to have complementary of results. Indeed, the antioxidant reaction mechanisms in the different tests are variable, and ideally an antioxidant should respond positively to different mechanisms. In this context, the antioxidant power of *L. nobilis* and *A. unedo* was evaluated by four tests, namely hydroxyl radical, superoxide radical, lipid peroxidation, and DPPH radical scavenging activity. In fact, the ability to prevent lipid peroxidation could only be determined in lipophilic samples, including essential oils [29].

From our results, it was clear that AUEO and LNEO possess important antioxidant characteristics and, therefore, could be considered as a promising source of natural antioxidants. This capacity of *A. unedo* leaf extracts has already been reported by other authors [29,73].

It is well known that the antioxidant effect of an extract or compound is generally associated with their redox properties, allowing them to act as reducing agents [74]. This significant antioxidant activity of both plants could be attributed to the presence of antioxidant compounds. Some monoterpenes and oxygenated sesquiterpenes have been reported to have an inhibitory oxidation power [75]. Thus, the antioxidant activity of AUEO and LNEO might be attributed to the presence of high concentrations of, mainly, eucalyptol (1,8-cineole) (36.40%), linalool (10.34%), α-terpinen-4-ol (13.05%), α-terpinyl acetate (10.61%) for LNEO, and (+)-isomenthone (4.23%), α-terpineol (7.8%), and myristic acid (3.96%) for AUEO. Our findings are similar to those reported by previous studies on the antioxidant activity of AUEOs and LNEOs [29,48,76,77]. Hence, these results confirm the role of EOs as natural antioxidants as well as their natural protective role for human health to preserve many physiological functions.

### 4.3. Anti-Diabetic Activity

The evaluation of the anti-diabetic activity of both selected plants was carried out by testing their capacity to inhibit the activity of two carbohydrate-hydrolyzing enzymes (α-amylase and α-glucosidase) and also that of lipase, allowing them to slow down the absorption of fatty acids and, subsequently, induce the degradation of internal glucose.

From our results, essential oils obtained from *A. unedo* and *L. nobilis* showed remarkable anti-diabetic activity against the three selected enzymes. Furthermore, it has been reported that *A. unedo* aqueous extract exhibits a potent inhibitory effect against α-amylase and α-glucosidase activity with IC_50_ values of 730.15 ± 0.25 and 94.81 ± 5.99 μg/mL, respectively [78].

On the other hand, previous studies have reported that LNEO possesses a potent inhibitory effect of more than 90% on α-glucosidase inhibitory activity, with an IC_50_ value of 1.748 ± 0.021 μL/mL [48]. Thus, this supports our suggestion that AUEO and LNEO have significant anti-hyperglycemic activities.

Based on the results of the lipase test and the fact that anti-diabetic activity was found, it seems likely that AUEO and LNEO could be natural sources of agents that stop fat from being absorbed and could be used to treat obesity. Despite numerous studies on the chemical composition and antimicrobial activity of AUEOs and LNEOs, there is little information on their anti-diabetic activity. In this study, our results revealed that both EOs exhibit high inhibitory activities towards α-amylase, α-glucosidase, and pancreatic lipase. This interesting anti-hyperglycemic effect may be due to several bioactive compounds, in particular those with high concentrations. Previous work showed that 1,8-cineole, α-pinene, and limonene inhibit α-glucosidase and α-amylase activities [48,79,80]. The combination of limonene and linalool has been reported to provide potent anti-hyperglycemic activity [81]. Other studies have revealed that terpinen-4-ol has remarkable anti-diabetic activity [75]. On the other hand, and as mentioned above, the most abundant volatile components in *A. unedo* were (+)-isomenthone, α-terpineol, and myristic acid and therefore the inhibition of the enzymes could be related to the presence of these components in the EOs. Moreover, AUEOs showed a highly significant correlations between their bioactive compounds and the percentage of α-glucosidase inhibition [82]. Similarly, the in vitro inhibitory activity of LNEOs against pancreatic lipase might be due to their volatile compounds. Our results are promising and suggest that EOs may be able to treat diabetes, but more research is needed to prove this assumption.

### 4.4. Anti-Acetylcholinesterase Activity

AChE is an important pathogenic factor in AD and its main role is to stimulate the hydrolysis of acetylcholine (ACh) to choline. Indeed, ACh deficiency is responsible for AD pathogenesis [83]. Several pharmacological investigations have focused on AChE inhibitors to reduce cholinergic deficits and improve neurotransmission [84]. However, these inhibitors have certain limitations, such as their short half-lives and the associated hepatotoxicity, which represents the main side effect [85]. This prompted several researchers to develop new anti-AChE agents [86].

In our study, EOs from *A. unedo* and *L. nobilis* showed significant anti-AChE activity that could be associated with their main compounds. Indeed, other studies have already shown that terpenes have anti-cholinesterase properties [87,88,89,90]. The mechanisms of action often involve competitive inhibition of the enzyme following the binding of bioactive molecules to the active site of the enzyme [91,92].

### 4.5. Anti-Inflammatory Activity

Inflammation is a natural response of the immune system, characterized by a mechanism that represents a chain of organized and dynamic responses comprising both cellular and vascular events with specific humoral secretion. These pathways involve physical changes in the localization of white blood cells (monocytes, basophils, eosinophils, and neutrophils), plasma and fluids at the inflamed site [93]. It is characterized by leukocyte activation, increased vascular permeability, edema, and pain [94].

The results of our tests (both in vivo and in vitro) show that the EOs we obtained from the leaves of *A. unedo* and *L. nobilis* have strong anti-inflammatory properties. These effects could be attributed to the presence of various bioactive molecules such as eucalyptol (1,8-cineole), α-terpinen-4-ol, camphor, α-terpinyl acetate, linalool, limonene, α-pinene, and camphene. Indeed, AUEO showed less 5-LOX inhibitory activity compared to LNEO. Moreover, AUEO exhibited remarkable activity (IC_50_ = 86.14 ± 0.05 μg/mL), which may be due to the presence of certain chemical compounds.

Furthermore, AUEO contains other molecules such as geraniol, which have already demonstrated a significant inhibitory effect, inhibiting pro-inflammatory cytokines as well as NF-κB signaling pathways [95]. In another study, Su and his colleagues [96] found that geraniol has a promising effect on nitric oxide and prostaglandin E2 (PGE_2_), which are pro-inflammatory molecules. On the other hand, previous studies have reported the anti-inflammatory effects of *A. undo* extracts [97,98]. However, the mechanisms underlying this effect have yet to be clarified.

Eucalyptol was reported as a major compound of LNEO (36.40%), and may provide gastroprotection via anti-inflammatory mechanisms; it could possess an inhibitory activity against 5-LOX, one of the key mediators involved in inflammatory responses [99]. Moreover, eucalyptol in interaction with limonene has been shown to cause partial potentiation of anti-inflammatory action [100]. In fact, limonene has been shown to inhibit lipopolysaccharide-induced inflammation and inflammatory cell migration [99].

Furthermore, monoterpenoid compounds have demonstrated potential anti-inflammatory effects. Indeed, several studies have shown that linalool is an anti-inflammatory agent, controlling the release of anti-inflammatory cytokines and regulating the activation of transcription factor-κB (NF-κB) and its translocation in the nucleus [101]. Similarly, camphene appears to be responsible for inhibiting the incorporation of arachidonic acid into the active site of the corresponding enzymes, thus inhibiting the production of inflammatory prostaglandins and leukotrienes [32].

Therefore, LNEO contains interesting bioactive compounds responsible for anti-inflammatory activities. Indeed, several studies have examined the effectiveness of LNEO as an effective anti-inflammatory agent [102,103,104]. Our results showed that LNEO compounds can be effective at inhibiting 5-LOX.

## 5. Antimicrobial Activity

In recent years, antibiotic resistance has become one of the most serious threats to global health, food security, and development. It is a natural phenomenon, but the misuse of antibiotics in humans and animals accelerates the process. The search for anti-microbial agents of natural origin will therefore provide a promising alternative. As has already been indicated, the tested EOs exerted significant anti-microbial activities against the different microorganisms.

Indeed, there have been many scientific publications on medicinal plant research indicating the remarkable sensitivity of Gram-positive bacteria to plant extracts or EOs compared to Gram-negative bacteria, which can initially be attributed to the cell wall structure [13,105,106]. Some previous studies have reported that AUEO has remarkable antimicrobial activity. It has been published that the methanol, ethanol, and ethyl acetate extracts of *A. unedo* leaves exhibit different levels of antibacterial activity against *E. coli*, *S. aureus*, *L. monocytogenes*, and *P. aeruginosa* strains that cause food-borne diseases; however, only the methanol extract showed antibacterial activity against *P. aeruginosa* with no effect from the ethanol and ethyl acetate extracts [107]. This is in harmony with the current results, which showed that *P. aeruginosa* was the least susceptible bacterium to the tested EOs. On the other hand, a previous study showed that the fruits, twigs, and leaves of *L. nobilis* recorded good antimicrobial activity against almost all microorganisms tested, including Gram-positive and Gram-negative bacteria, yeasts, and fungi; however, *P. aeruginosa* showed the lowest response [69]. Indeed, *P. aeruginosa* is a difficult-to-treat microorganism. It is highly resistant to numerous antibiotics, including ciprofloxacin, gentamicin, tobramycin, imipenem, and ceftazidime [108]. The general resistance of *P. aeruginosa* is well documented, and its genes can express a wide range of resistance mechanisms; mutations in chromosomal genes that control resistance genes can occur, and it can acquire resistance genes from other organisms through plasmids, transposons, and bacteriophages [109]. Therefore, *P. aeruginosa* was classified by the WHO as a resistant pathogen on a list of antibiotic-resistant bacteria that are in urgent need of new alternative treatments [110].

Our findings regarding MIC and MLC are consistent with previously published reports, suggesting that EOs from these plants can be used as nutraceuticals and functional foods as well as antimicrobial agents, The MIC and MBC of the leaves of *Laurus nobilis* was reported to be as low as 2.5 g/L for *Escherichia coli* and 1.25 g/L (MIC) and 2.5 g/L (MBC) for *Yersinia enterocolitica*, where the *Arbutus Unedo* root extracts showed relatively high MIC compared with *Laurus nobilis* leaves against *S. aureus* (MIC = 12.5 mg/mL) [111,112,113,114]. Essential oils mostly have higher inhibitory activity against microorganisms than crude extracts as they have more aromatic compounds with antimicrobial effects [115].

Overall, the findings of our current investigation are consistent with those of previous publications using the diffusion method that showed potent broad-spectrum antibacterial ability [116,117]. It is claimed that the mode of action of the EOs against target microorganisms is different from the regular antibiotics; when exposed to EOs, bacterial cells might be destroyed by the irregular disruption of the intracellular structure and the bursting of cell walls and membranes [105]. This aspect needs much future study, since so far medicinal plants have not been used as a drug alternative to antibiotics.

## 6. Conclusions

Here, we currently report the identification of phytochemicals as well as some biological properties of AUEO and LNEO. Phytochemical analysis revealed a diversity of volatile compounds in both EOs. The results showed significant antioxidant, anti-diabetic, and antimicrobial effects with very low EO concentrations. Other results are needed to highlight the mechanisms by which these EOs act. The major compounds of these oils must also be investigated for their biological effects. In addition, toxicity studies should be performed to verify the safety of EOs and bioactive compounds.

## Figures and Tables

**Table 1:** Chemical composition of AUEO and LNEO.

Number	AUEO			LNEO		
	R_T_	Compounds	%	R_T_	Compounds	%
**1**	0.479	Caryophyllene	0.26	2.036	*α*-Thujene	0.43
**2**	0.805	Myrtenol	0.78	2.115	*α*-Pinene	3.34
**3**	1.481	Geraniol	0.57	2.262	Camphene	0.47
**4**	1.741	*β*-Eudesmol	1.28	2.791	*β*-Thujene	5.74
**5**	2.439	*γ*-Eudesmol	1.43	4.132	Eucalyptol	36.40
**6**	3.228	Nonanoicacid	4.38	5.947	Linalool	10.34
**7**	4.445	Decenal	13.47	7.479	*α*-terpineol	13.05
**8**	5.099	Palmitic acid	6.00	10.048	4-Thujen-2-α-yl acetate	0.91
**9**	5.211	(E,Z)-2,6-Nonadienal	0.62	10.150	Bornyl acetate	0.87
**10**	5.420	(E)-2-Undecenal	0.7	12.426	*α*-Terpinyl acetate	10.61
**11**	8.186	(E)-Geranylacetone	3.36	12.595	Eugenol	1.58
**12**	10.057	*α*-Terpineol	7.8	14.071	Methyleugenol	3.74
**13**	10.125	Linalool	1.82	15.018	Naphthalene	0.64
**14**	10.226	Nonanal	3.8	17.441	*β*-Neoclovene	0.50
**15**	10.384	Dodecanoicacid	1.2	17.666	Isoelemicin	0.38
**16**	11.409	*β*-Ionone	1.26	-	-	-
**17**	11.815	Octanol	0.64	-	-	-
**18**	17.167	Myristic acid	3.96	-	-	-
**Total identified compounds %**			53.33%	**Total identified compounds %**		89%
**Monoterpene hydrocarbons %**			-	**Monoterpene hydrocarbons %**		4.88%
**Oxygenated monoterpenes %**			24.44%	**Oxygenated monoterpenes %**		72.18%
**Sesquiterpene hydrocarbons %**			1.52%	**Sesquiterpene hydrocarbons %**		6.24%
**Oxygenated sesquiterpenes %**			2.71%	**Oxygenated sesquiterpenes %**		0.38%

R_T_: retention time.

**Table 2:** Antioxidant activity of AUEO and LNEO.

EOs	Hydroxyl IC_50_ (μL/mL)	Superoxide IC_50_ (μL/mL)	Lipid Peroxidation IC_50_ (μL/mL)	DPPH IC_50_ (μL/mL)
***Laurus nobilis***	0.354 ± 0.02 ^a^	0.133 ± 0.01 ^a^	0.101 ± 0.05 ^a^	0.489 ± 0.07 ^a^
*** Arbutus unedo ***	0.527 ± 0.01 ^b^	0.275 ± 0.07 ^b^	0.207 ± 0.03 ^b^	0.711 ± 0.04 ^b^
** BHT **	12.027 ± 0.01 ^c^	43.307 ± 0.001 ^c^	2.022 ± 0.031 ^c^	21.057 ± 0.051 ^c^

Different letters in the same column represent significant differences at *p* < 0.05.

**Table 3:** The inhibition of the digestive enzymes α-glucosidase, α-amylase, and lipase by AUEO and LNEO.

IC_50_ (µg/mL)	α-Amylase	α-Glucosidase	Lipase
*Arbutus unedo* EO	102 ± 0.06 ^c^	76 ± 0.021 ^c^	97.018 ± 0.012 ^c^
*Laurus nobilis* EO	42.51 ± 0.012 ^b^	1.347 ± 0.021 ^a^	21.23 ± 0.021 ^b^
Acarbose	32.14 ± 0.016 ^a^	22 ± 0.005 ^b^	_
Orlistat	_	_	14.12 ± 0.023 ^a^

Different letters in the same column represent significant differences at *p <* 0.05. AUEO: *Arbutus unedo* EO. LNEO: *Laurus nobilis* EO.

**Table 4:** AChE inhibitory activities of EOs compared to the standard drug rivastigmine.

IC_50_ (µg/mL ± SEM)	AUEO	LNEO
AChE	378.57 ± 0.05 ^b^	89.44 ± 0.07 ^a^
Rivastigmine	-	2.24 ± 0.03 ^c^

Different letters in the same column represent significant differences at *p <* 0.05. AUEO: *Arbutus unedo* EO. LNEO: *Laurus nobilis* EO.

**Table 5:** In vitro anti-inflammatory activity of AUEO and LNEO.

Assays.	(IC_50_ μg/mL)		Control
	AUEO	LNEO	Quercetin
5-Lipoxygenase	86.14 ± 0.05 ^c^	48.31 ± 0.07 ^b^	17.59 ± 0.01 ^a^

Different letters in the same column represent significant differences at *p* < 0.05. AUEO: *Arbutus unedo* EO. LNEO: *Laurus nobilis* EO.

**Table 6:** Inhibition percentage of the left hind paw volume in rats treated with AUEO and LNEO.

Compounds	Dose (mg/kg)	Carrageenan-Induced Hind Paw Edema Volume (mL; Mean) and % of Inhibition						
		T_0_	1 h	% inh.	3 h	% inh.	6 h	% inh.
Control	-	0.84	1.43	-	1.63	-	1.86	-
*L. nobilis*	50	0.89	1.31	28.81	1.27	51.90	1.19	70.59
	100	0.89	1.23	42.37	1.15	67.09	1.01	88.26
*A. unedo*	50	0.76	1.24	18.64	1.25	37.97	1.18	58.82
	100	0.82	1.26	25.42	1.23	48.10	1.19	63.72
Indomethacin	10	0.85	1.14	50.85	1.16	60.76	1.13	72.55

**Table 7:** The antimicrobial activities of *A. unedo* and *L. nobilis* EOs assessed using disc diffusion test (diameter equals 6.0 mm means no inhibition).

Microorganism	*Arbutus unedo* EO (100%)	*Laurus nobilis* EO (100%)	Chloramphenicol (30 µg/mL)	Nystatin (100 I.U.)
*Escherichia coli* ATCC 25922	14.6 ± 0.2	16.2 ± 0.1	22.9 ± 0.1	0.0
*Proteus mirabilis* ATCC 25933	14.2 ± 0.1	15.6 ± 0.2	22.6 ± 0.2	0.0
*Salmonella typhimurium* ATCC 700408	11.0 ± 0.1	12.7 ± 0.8	13.6 ± 0.0	0.0
*Pseudomonas aeruginosa* ATCC 27853	8.0 ± 0.0	8.0 ± 0.0	6.0 ± 0.0	0.0
*Bacillus subtilis* ATCC 6633	16.2 ± 0.3	18.0 ± 0.2	16.3 ± 0.1	0.0
*Staphylococcus aureus* ATCC 29213	15.4 ± 0.2	18.3 ± 0.2	25.6 ± 0.1	0.0
*Listeria monocytogenes* ATCC 13932	16.9 ± 0.2	19.3 ± 0.2	28.6 ± 0.2	0.0
Candida albicans	16.2 ± 0.2	19.8 ± 0.3	0.0	28.8 ± 0.3
*Trichophyton rubrum*	13.0 ± 0.3	15.6 ± 0.3	0.0	25.0 ± 0.02
*Aspergillus niger*	13.4 ± 0.2	16.2 ± 0.2	0.0	25.8 ± 0.1

Diameter equals 6.0 mm means no inhibition, mean ± standard deviation.

**Table 8:** MIC and MLC values of AUEO and LNEO.

Microorganisms	EOs % (*v*/*v*)				Controls (µg/mL)	
	AUEO		LNEO		Chloramphenicol	Nystatin
	MIC	MLC	MIC	MLC	MIC	MIC
*Escherichia coli* ATCC 25922	1.0	2.0	1.0	1.0	4.0	NT
*Proteus mirabilis* ATCC 25933	2.0	2.0	1.0	1.0	4.0	NT
*Salmonella typhimurium* ATCC 700408	2.0	4.0	1.0	2.0	64.0	NT
*Pseudomonas aeruginosa* ATCC 27853	>4.0	>4.0	>4.0	>4.0	>64.0	NT
*Bacillus subtilis* ATCC 6633	1.0	2.0	0.5	1.0	32.0	NT
*Staphylococcus aureus* ATCC 29213	1.0	2.0	0.5	1.0	4.0	NT
*Listeria monocytogenes* ATCC 13932	1.0	1.0	0.5	1.0	2.0	NT
*Candida albicans*	0.5	NT	0.25	NT	NT	4.0
*Trichophyton rubrum*	2.0	NT	1.0	NT	NT	16.0
*Aspergillus niger*	2.0	NT	1.0	NT	NT	16.0

NT: not tested.

## Data Availability

Not applicable.

## Abbreviations

ABTS	2,2-azino-bis-3-ethylbenzothiazoline-6-sulfonic acid
Ach	Acetylcholine
AChE	Acetylcholinesterase
AD	Alzheimer’s Disease
ASCh	Acetylthiocholine Iodide
BHT	Butylated Hydroxytoluene
AUEO	*Arbutus unedo* Essential Oil.
DM	Diabetes Mellitus
DMSO	Dimethyl Sulfoxide
DNA	Deoxyribonucleic Acid
DPPH	2,2-diphenyl-1-picrylhydrazyl
DTNB	5,5′-dithio-bis-2-nitrobenzoic acid
EO	Essential Oil
EDTA	Ethylenediaminetetraacetic acid
FeCl_3_	Chlorure ferrique
GC-MS	Gas Chromatography-Mass Spectrometry
LNEO	*Laurus nobilis* Essential Oil.
MHA	Mueller-Hinton Agar
MFC	Minimum Fungicidal Concentration
MIC	Minimum Inhibitory Concentration
MLC	Minimum Lethal Concentration
NBT	Nitro Blue Tetrazolium
NF-κB	Transcription Factor-κB
OH	Hydroxyl Radical
PGE_2_	Prostaglandin E_2_
R_T_	Retention Time
SA	Sabouraud dextrose agar
TBA	Thiobarbituric Acid
TBARS	Thiobarbituric Acid Reactive Substances
TCA	Trichloroacetic Acid
Trolox	6-hydroxy-2,5,7,8-tetramethylchroman-2-carboxylic acid
WHO	World Health Organization
5-LOX	Lipoxygenase
TBA	Thiobarbituric Acid
